# Evaluating the Effectiveness and Safety of Home Facial Antiaging Beauty Devices Based on Meridian and Acupoint Theory

**DOI:** 10.1111/jocd.70096

**Published:** 2025-03-18

**Authors:** Pengzhi Bu, Ji Luo, Chuanbiao Wen, Jing Xu, Guangtao Pan

**Affiliations:** ^1^ Institute of Intelligent Medicine, Chengdu University of Traditional Chinese Medicine Chengdu Sichuan China; ^2^ College of Acupuncture and Massage Chengdu University of Traditional Chinese Medicine Chengdu Sichuan China; ^3^ Yancheng TCM Hospital Affiliated to Nanjing University of Chinese Medicine Yancheng Jiangsu Province China

**Keywords:** efficacy, home antiaging facial beauty devices, meridian and acupoint theory, physical improvement, safety

## Abstract

**Background:**

The increasing use of home beauty devices for antiaging raises questions about their efficacy and safety. Traditional Chinese medicine suggests that stimulating certain meridians and acupoints can aid in antiaging. This study evaluates the effects of two popular home facial devices on facial and body aging, integrating meridian and acupoint theory.

**Methods:**

A randomized controlled trial with 90 volunteers (25–65 years) over 4 weeks assessed the devices' impact on skin aging and physical health. Participants were divided into three groups: Group Y, Group J, and a control group. Outcomes were measured using VISIA imaging, standardized photographs, and the Quality of Life Scale, with safety assessments included.

**Results:**

Of the 90 participants, 80 completed the study. Both Group J and Group Y showed significant improvements in skin wrinkles, texture, radiance, and laxity compared to the control group (*p* < 0.05). No significant differences were found in physical health improvements (*p* > 0.05).

**Conclusion:**

The devices showed effectiveness in improving facial skin aging but require further safety verification. The potential of meridian theory integration should be explored further, with a focus on device design and user‐friendliness for future research.

## 1 Introduction

In recent years, home beauty devices have garnered significant consumer interest due to their low cost, portability, and customizable features. The effectiveness and safety of these devices have become a focal point of public concern. The beauty instruments available on the market primarily utilize technologies such as laser, radiofrequency (RF), microcurrent, LED, and intense pulsed light (IPL) [1], which can be used individually or in combination to achieve enhanced aesthetic results. However, some dermatologists have noted that compared to medical devices, home beauty devices have lower energy output, leading to less effective results [2].

Radiofrequency technology works by applying high‐frequency currents to the skin tissue, generating a significant thermal effect due to the skin's inherent impedance [3]. This localized increase in temperature promotes changes in the collagen structure, stimulating fibroblasts to synthesize new collagen and elastin fibers. Post‐RF treatment, there is a significant increase in the levels of heat shock proteins 72 (HSP72), transforming growth factor‐β, and vascular endothelial growth factor, among other biomarkers, which contribute to skin firming and reduction of wrinkles [4, 5, 6]. Photobiomodulation activates the respiratory pathways of cellular mitochondria, promoting fibroblast proliferation, collagen synthesis, and the production of growth factors and extracellular matrix [7]. Microcurrents accelerate the repair process of the dermis and subcutaneous connective tissue; their electrical stimulation is compatible with the body's natural electrical currents and positively affects fibroblast proliferation, neovascularization, and epithelial layer thickening, leading to skin firming and wrinkle reduction [8].

According to the meridian theory of traditional Chinese medicine, acupoints are considered key reactive points within the human meridian system. Moderate stimulation of these acupoints can regulate the balance of Qi and blood, promote blood circulation, enhance cellular vitality, and thereby delay the aging process and alleviate aging‐related symptoms [9]. For instance, acupuncture at acupoints such as Dicang (ST4), Taiyang (EX‐HN5), and Yangbai (GB14) has been proven to effectively improve the firmness, elasticity, and luster of facial skin [10]. Moreover, the Dicang acupoint is closely connected with facial muscles, nerves, and visceral organs such as the digestive system. Stimulating this acupoint can activate the nervous system, promote nerve conduction, and improve neurofunctional disorders [11]. In recent years, the application of physical stimuli such as microcurrent and LED technology in meridian‐acupoint therapy has also shown positive effects [12, 13, 14].

This study, grounded in meridian theory, aims to evaluate the effectiveness of two home facial antiaging beauty devices, compare their antiaging efficacy and safety, and explore their impact on facial meridians and acupoints to achieve antiaging effects. To this end, we conducted a 4‐week randomized controlled experiment.

In the experiment, we tested two home antiaging beauty devices centered around radiofrequency, microcurrent, and LED technology. These devices differ in energy output and exterior design (Table 1). They are designed to improve wrinkles and enhance facial contours through varying intensities of radiofrequency, LED, and microcurrent. Additionally, the devices are equipped with temperature, current, and motion sensors to ensure safety during use, preventing skin discomfort or damage due to excessive temperature, high current, or prolonged use.

**TABLE 1:** Comparison of the appearance, function, main parameter settings, and safety functions of two household facial antiaging beauty devices.

	Ya‐Man ACE five generations (Ya‐Man Corporation, Japan)	Jmoon Speed beauty instrument (Jmoon Corporation, China)
Appearance design	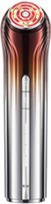	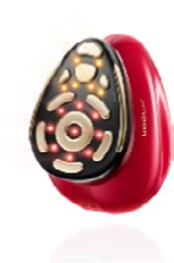
The claimed efficacy	Improve the forehead wrinkles、laxity around the eyes, sagging of the malar fat pads and a blurred jawline.	Lighten the nasolabial folds、tight the eye area and whole face, raised the jaw line.
Key parameter setting	Radiofrequency: 0.5–2.5 MHz LED: 630 ± 10 nm red light Microcurrent: slow‐release current	Radiofrequency: 0.8–2.0 MHz LED: 620‐630 nm red light and 580–590 nm orange light Microcurrent: Not mentioned
Safety function	Temperature sensor, current sensor, motion sensor	Temperature sensor, current sensor, motion sensor

## 2 Methods

### 2.1 Study Participants

This study has been registered as a clinical trial. A total of 90 Chinese volunteers were recruited for this study, with 80 completing all study procedures and 10 withdrawing due to personal reasons. Inclusion criteria comprised individuals aged between 25 and 60 years, in good general health; exhibiting signs of facial aging such as skin laxity and wrinkles; and willing to adhere to study requirements and complete treatments and follow‐ups. Exclusion criteria included individuals with a history of skin diseases like eczema or psoriasis; women who were pregnant or nursing; and those who had undergone facial rejuvenation procedures (such as botulinum toxin injections) or had acupuncture treatment within the past 3 months. All participants provided written informed consent.

All participants were tested and photographed under standardized environmental conditions with a temperature of 21°C ± 2°C and a relative humidity of 50% ± 10%. After cleansing their faces, participants were required to wait for 30 min to allow for subsequent testing and standardized photograph capture. During this waiting period, participants completed the Quality of Life Questionnaire.

### 2.2 Materials

During the experimental process, participants in Group Y used the YA‐MAN device, while those in Group J used the Jmoon device. The control group was assigned to use a non‐powered radiofrequency beauty device. All treatment procedures utilized a medical coupling agent (Hainuo Biotech, Qingdao, China) to enhance conductivity, reduce friction, and ensure the convenience and safety of the treatment.

### 2.3 Study Design

**FIGURE 1:**
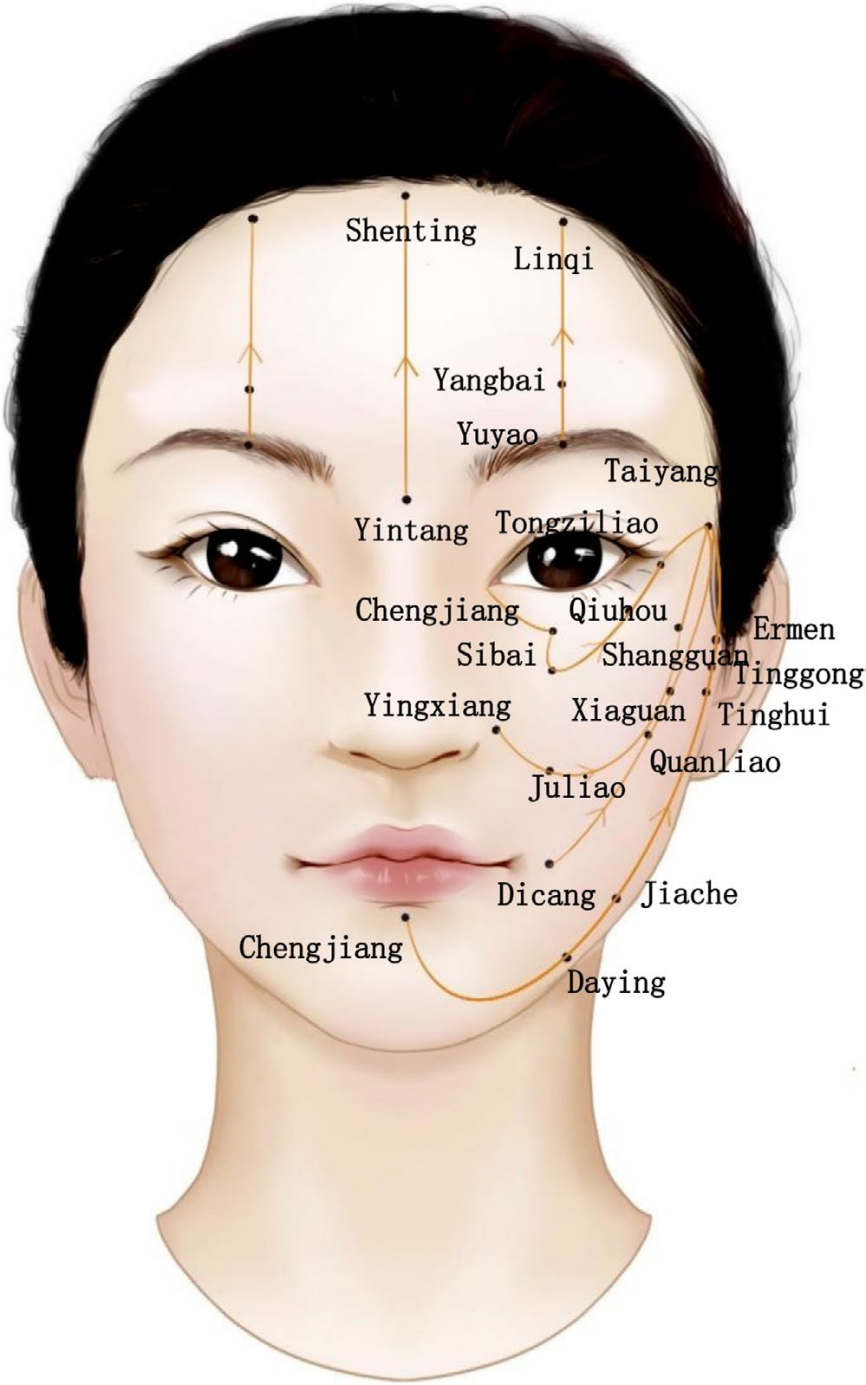
In accordance with meridian and acupoint theory, this study employs a diagrammatic method to treat each side of the subjects' faces for 3 min, a treatment process that simultaneously stimulates facial meridians and acupoints.

This study was a 4‐week randomized controlled trial. Participants were randomly assigned to three different groups. Based on the random allocation results, researchers selected the appropriate devices and followed specific treatment techniques that integrated traditional Chinese medicine meridian theory (Figure 1). Each participant's face was treated for 3 min per side and 1 min on the forehead, totaling 7 min. During the treatment, researchers adjusted the device settings based on real‐time feedback from the participants. The devices were used five times per week. Participants maintained their regular skincare products and routines throughout the trial. At designated time points, the participants' skin biophysical properties and physical condition improvements were assessed.

### 2.4 Objective Assessment

The study utilized the VISIA‐CR skin analysis imaging system (Canfield Imaging Systems, Fairfield, NJ, USA) to capture images of participants' frontal and left and right 45° angle views. To ensure consistency in results, the photography and lighting parameters, as well as the distance between the face and the lens, were maintained uniformly for all participants at different time points.

### 2.5 Clinical Evaluation

At the initial stage (T0) and the final stage (T1) of the study, standardized facial photographs of the participants were taken by a professional photographer using a digital camera under uniform lighting and background conditions. The photographs included frontal views and 45° lateral views to ensure comprehensive documentation. The evaluation covered wrinkles (overall facial wrinkles, periorbital wrinkles, perioral wrinkles, nasolabial folds, forehead wrinkles, and tear troughs), laxity (mandibular angle and cervicomental angle), and skin condition (firmness, luster, and pigmentation).

Two dermatologists with extensive clinical experience, who were not involved in the study intervention process, assessed the photographs. These evaluators underwent specific training in testing and scoring methods. They referenced the Wrinkle Severity Rating Scale (WSRS) and the Merz Jawline Assessment Scale (MJAS) for standardized evaluation criteria. The Global Aesthetic Improvement Scale Score (GAIS Score) was used to assess the standardized photographs taken at T0 and T1. The GAIS Score ranges from 0 to 4, where 0 indicates a worse overall condition compared to baseline, 1 indicates no change, 2 indicates slight improvement, 3 indicates noticeable improvement, and 4 indicates highly significant improvement.

### 2.6 Safety Evaluation

Researchers will document any adverse reactions experienced by each participant during the treatment and follow‐up periods. These reactions include, but are not limited to, postoperative erythema, edema, pigmentation, pain, peeling, and scarring.

### 2.7 Self‐Assessment

At the end of the 4‐week treatment period, all subjects were required to complete a satisfaction survey questionnaire to self‐assess their overall feelings about the treatment. The questionnaire assessed various dimensions, including treatment effects and treatment experience. Self‐assessment used a 5‐point rating system, with 1 indicating the lowest satisfaction and 5 representing the highest satisfaction, with higher scores indicating greater satisfaction among the subjects.

### 2.8 Health Status Improvement

Based on meridian and acupoint theory, the study selected seven key indicators from the Quality of Life Scale: health status, sleep quality, appetite, vitality, negative emotions, fatigue, and memory. Participants assessed these indicators at T0 (baseline) and T1 (post‐intervention) according to their actual conditions, using a 1–5 scoring system. In the scoring system, high scores for health status, sleep quality, appetite, and vitality indicate better health status, whereas low scores for negative emotions, fatigue, and memory indicate better health status.

### 2.9 Data Analysis

Data in this study were analyzed using statistical methods. For data that conform to a normal distribution, we used paired sample *t*‐tests or two independent sample *t*‐tests; for data that do not conform to a normal distribution, the Wilcoxon signed‐rank test was used. For subjective assessment indicators, if the evaluation criteria of the two doctors were consistent, we used the mean ± standard deviation of either doctor's score to represent the improvement in subjective assessment indicators. All statistical analyses used a *p* value less than 0.05 as the criterion for determining the presence of significant differences.

## 3 Results

### 3.1 Objective Evaluation

**FIGURE 2:**
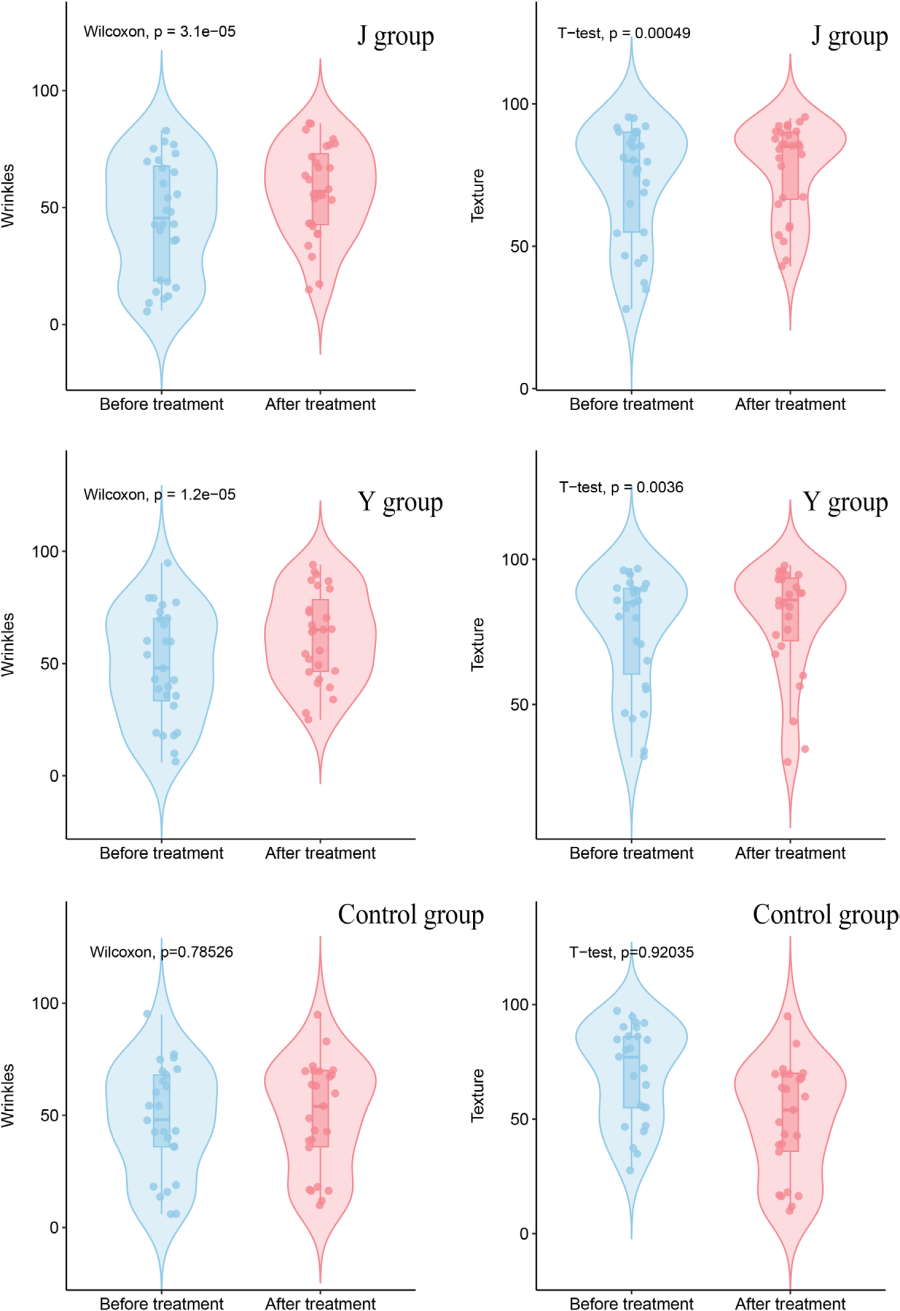
The results of the difference test for wrinkle and texture scores between Group Y, Group J, and the control group at T0 and T1 time points. There is a significant difference in wrinkle and texture scores between the two time points for both Group Y and Group J (*p* < 0.05), while the control group shows no significant difference in scores before and after the intervention.

At T0 and T1 time points, we conducted normality tests on the wrinkle and texture scores of the subjects in the three groups. For texture characteristic data, which conformed to a normal distribution, we employed the *t*‐test for evaluation; whereas for wrinkle characteristic data, which did not conform to a normal distribution, we utilized the Wilcoxon signed‐rank test for assessment. Comparisons between T0 and T1 for Group J and Group Y showed significant differences (*p* < 0.05), indicating statistical significance. In contrast, the differences before and after the intervention for the control group were not significant (*p* > 0.05), indicating no statistical significance (Figure 2). These results suggest that the devices used by Group J and Group Y both had an improving effect on wrinkles and texture, while the control group did not demonstrate a noticeable intervention effect.

### 3.2 Clinical Evaluation

**FIGURE 3:**
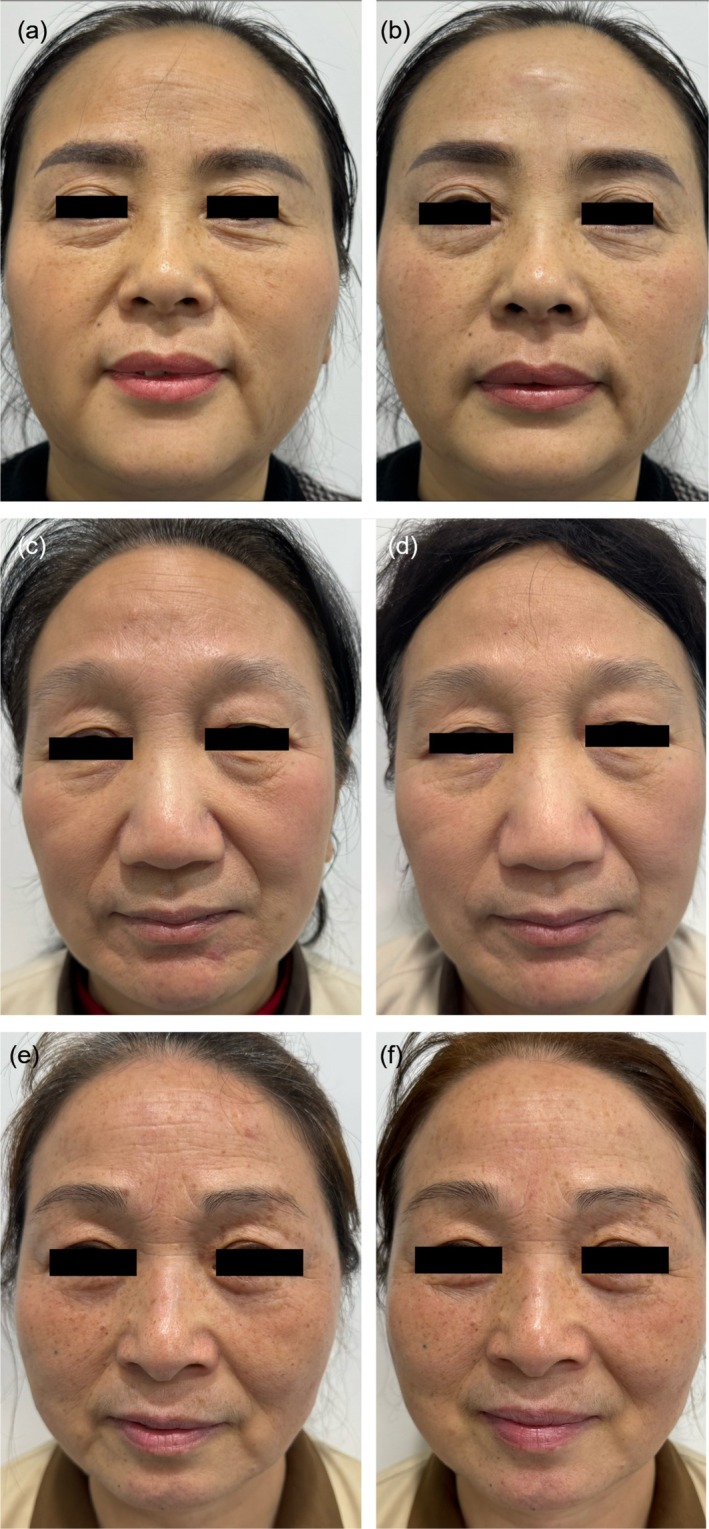
Standardized photographs of three subjects at T0 and T1 time points, where subject a is from Group Y, subject c is from Group J, and subject e is from the treatment group. It can be observed that both Group Y and Group J exhibit noticeable improvements in facial wrinkles and other aspects.

Figure 3 illustrates the standardized clinical photographs of three participants, aged between 48 and 50 years, at both the baseline (T0) and follow‐up (T1) time points across the three study groups. This visual representation allows for an assessment of the comparative efficacy of the interventions by examining the changes in facial features such as wrinkles, skin texture, and so forth between the initial and subsequent evaluations.

**FIGURE 4:**
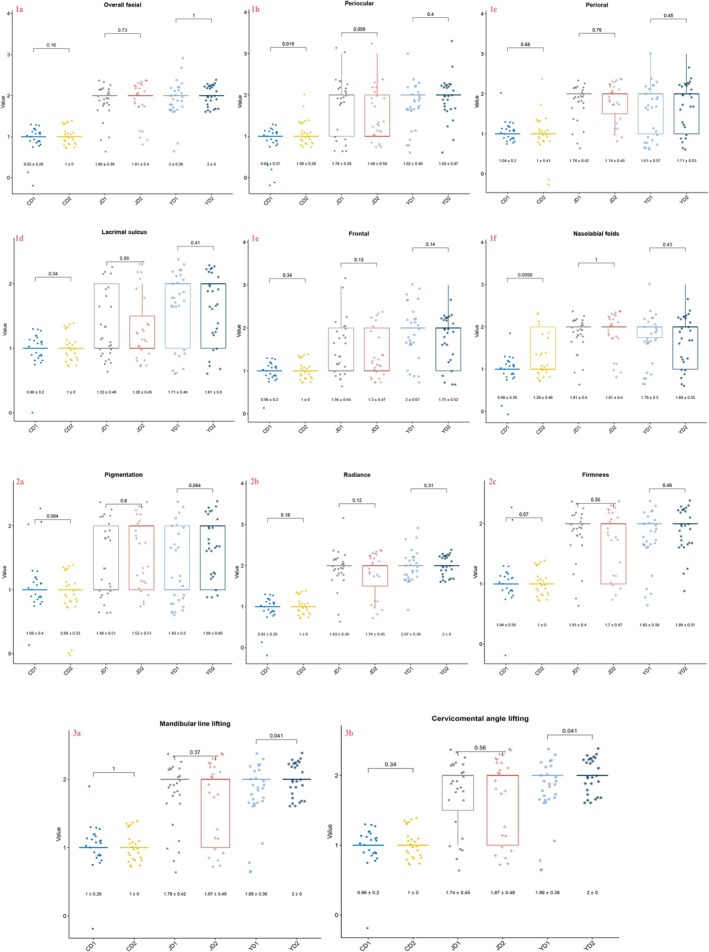
This figure delineates the comparative efficacy of two home antiaging devices (Group J and Group Y) against a control group (CD) across various facial regions. Panels 1a–1f illustrate that both devices exhibit improvements in overall facial, periocular, perioral, nasolabial folds, forehead wrinkles, and tear troughs compared to the control. Notably, Group J demonstrates superior efficacy in treating perioral and tear trough wrinkles, while Group Y shows better results in other assessed areas. Panels 2a–2c reveal that both Group J and Group Y enhance skin firmness, radiance, and pigmentation, with Group Y achieving marginally greater improvements. Panels 3a and 3b indicate that, according to the dermatologists' evaluations, Group Y outperforms Group J in lifting the cervicomental angle and mandibular line. In all referenced panels, CD denotes the control group, JD denotes Group J, and YD denotes Group Y.

At T0 and T1 time points, the two dermatologists had divergent opinions on the changes in periocular wrinkles and tear troughs for the control group, with statistically significant differences (*p* < 0.05). The differences in other scoring areas were not significant (*p* > 0.05), allowing the use of the mean ± standard deviation of either doctor's scores to represent the improvement effects for each region. Compared to the control group, both devices demonstrated improvement effects in wrinkle treatment. Notably, Group J showed superior treatment effects on perioral and tear trough wrinkles compared to Group Y; however, for overall facial wrinkles, periocular wrinkles, nasolabial folds, and forehead wrinkles, the effects of the two devices were reversed (Figure 4 1a–1f).

Regarding skin indicators, the *p* values for all groups were greater than 0.05, indicating that the assessments of the two doctors on firmness, radiance, and pigmentation were largely consistent and not statistically significant. Based on the mean values and standard deviations, both doctors unanimously agreed that the use of beauty devices had an improving effect on skin indicators, with Group Y showing a slightly higher improvement than Group J, although the difference was minimal (Figure 4 2a–2c).

For laxity indicators, the two doctors had inconsistent opinions on the changes in the jawline and submental angle for Group Y before and after the intervention, with statistically significant differences (*p* < 0.05), while the *p* values for other group indicators were all greater than 0.05, indicating no statistical significance. This suggests that the two doctors agreed on the improvement effects of Group J on the jawline and submental angle compared to the control group. According to the mean values and standard deviations, both doctors believed that Group Y's improvement effects were superior to those of Group J (Figure 4 3a and 3b).

### 3.3 Safety Evaluation

During the treatment process, some subjects experienced mild to moderate erythema, which resolved within 30 min after the treatment ended. The incidence of mild to moderate erythema in Group J was 26.7%, and in Group Y it was 23.5%, while no mild to moderate erythema was observed in the control group. There was no significant difference in the incidence rate between Group J and Group Y (*p* > 0.05), but both groups showed statistically significant differences compared to the control group (*p* < 0.05).

### 3.4 Self‐Evaluation

After 1 month of treatment, the scores for treatment comfort among the three groups did not show statistically significant differences (*p* > 0.05). In terms of satisfaction with the treatment effects, there was no significant difference between Group Y and Group J (*p* > 0.05), but both groups showed statistically significant differences compared to the control group (*p* < 0.05).

### 3.5 Health Status Improvement Evaluation

Analyzing the data from the seven indicators filled out by the subjects in the Quality of Life Scale at T0 and T1, it was found that these indicators did not conform to a normal distribution. Therefore, the study employed the non‐parametric Wilcoxon signed‐rank test for analysis. According to the results, the P‐values for all indicators before and after the experiment were greater than 0.05, indicating no statistical significance, which suggests that these indicators did not show significant improvement before and after the experimental intervention.

## 4 Discussion

The safety and efficacy of home beauty devices have consistently been a focal point of research and discussion. In recent years, clinical studies on these devices have been conducted by researchers. Marc Cohen and colleagues performed a systematic review assessing the safety and efficacy of home skin devices [15]. However, due to the rapid pace of innovation and iteration in home beauty devices, relevant regulatory policies have not fully kept pace. For instance, LED devices are not subject to FDA regulations for medical device treatments [2]. Despite the issuance of the “Guidance for the Registration and Review of Radiofrequency Aesthetic Devices” in China, there remain some home beauty devices on the market that promote other energy output modes and avoid mentioning radiofrequency technology in an attempt to evade regulation. Therefore, it is necessary to conduct more in‐depth research on the safety and efficacy of home beauty devices. This study employed a randomized controlled trial, integrated with traditional Chinese medicine meridian theory, to explore the effects of home antiaging beauty devices on facial rejuvenation and physical condition improvement, as well as the safety of their use.

In this study, we adopted a randomized controlled trial design to validate the efficacy and safety of home‐use facial antiaging beauty devices. Most existing studies on home‐use beauty devices for facial rejuvenation are non‐randomized controlled experiments [16]. To address this gap, we randomly assigned participants into three groups: one group used the Ya‐Man home‐use facial antiaging beauty device, another group used the Jmoon device, and the third group used a power‐free RF beauty device as the control. This grouping strategy aims to comprehensively evaluate the differences in antiaging effects among various devices. During the experiment, we integrated the recommended usage methods of the Ya‐Man and Jmoon devices with traditional meridian‐acupoint theory to enhance the antiaging effects through a combination of traditional Chinese and Western medicine approaches (specific procedures are shown in Figure 1). To ensure standardization and safety, medical coupling gel was used, and the treatment time was strictly controlled at 3 min per side of the face and 1 min for the forehead.

The study results indicated that both devices demonstrated certain efficacy in improving wrinkles, texture, and laxity, but there were differences in their effects across different areas and indicators. Overall, the ACE Fifth Generation Golden Five‐Ring RF device from Ya‐Man showed slightly better improvement effects than the Jmoon Beauty Iron, although the differences were not substantial. In terms of safety, some participants experienced transient erythema, suggesting that further improvements in safety are still needed. Regarding health status improvement, no significant statistical differences were observed between the T0 and T1 assessments.

Table 1 lists the appearance and parameter settings of the two devices. Notably, the Jmoon device showed superior improvement effects on tear troughs and perioral wrinkles, which may be related to its design, as the smaller probe allows for more precise treatment of these areas. Additionally, research indicates that the ring‐shaped electrode used in the Yarman device is superior to strip or multi‐point electrodes in certain cases, as it can produce a more uniform heat distribution [17].

During the experimental process, this study did not use the gels provided with the two devices as conductors for treatment, but instead chose medical coupling agents to reduce the impact of gel ingredients on the experimental results. For instance, the gel provided with the Yarman device contains retinyl palmitate, which can promote collagen synthesis and reduce skin texture [18]; squalane not only inhibits the peroxidation of skin lipids, delays skin aging, but also enhances cellular metabolism, aids in repairing damaged cells, and achieves the purpose of repairing the skin barrier [19], which may to some extent, reduce the occurrence of erythema, allergies, and other safety issues during use.

In recent years, researchers have increasingly explored the potential of facial acupoint stimulation for facial rejuvenation [20]. Studies have shown that stimulating facial meridian acupoints can not only increase skin hydration and sebum content but also improve signs of facial skin aging. Additionally, the use of Juvederm Ultra Plus [21] and Avelan [22] for injections into facial acupoints such as Dicang (ST4) has been observed to significantly improve facial pigmentation, spots, swelling, skin texture, and luster.

From a biological perspective, stimulating meridian acupoints can activate the nervous system, trigger the release of immune cytokines, modulate the immune system, and enhance the body's defense against pathogens [23]. For instance, the acupoints Yintang (EX‐HN3) and Shenmen (HT7) have been proven to alleviate anxiety and sleep disorders [24, 25]. The Sibai (ST4) acupoint, which is believed to have beautifying effects in traditional meridian theory, has also been supported by modern research. It has been shown to regulate gastric function by activating medullary central nuclei neurons related to visceral information [26].

However, due to significant differences in microcurrent intensity or LED energy output between medical and home‐use devices, and the relatively weaker stimulation intensity on relevant acupoints, combined with the lack of more objective and precise methods for assessing health status, this study failed to demonstrate a significant improvement in participants' health status statistically. Future research could optimize study designs by randomly comparing the recommended usage methods of different devices with the operation methods used in this study to verify which experimental protocol is more effective, thereby providing more reliable evidence for the application of home‐use beauty devices in health improvement.

Although some physicians believe that home‐use beauty devices can serve as post‐treatment maintenance tools after medical aesthetic procedures, empirical data to support this notion are currently lacking. This study provides valuable insights into the efficacy of two beauty instruments. However, future research should further explore their long‐term effects and safety with larger sample sizes and longer follow‐up periods. As an emerging field, home‐use beauty devices need not only to be effective and safe but also convenient to use and suitable for various application scenarios, all of which require further optimization. For example, some studies have evaluated the ease of use and clarity of instructions in addition to safety and efficacy [27]. The application of software may help consumers implement more personalized treatments, such as selecting appropriate modes and enhancing user confidence, which also needs further investigation. Meanwhile, applying meridian‐acupoint theory to home‐use beauty devices can offer consumers multifunctional and personalized beauty solutions, bringing new possibilities and directions to the field of beauty and skincare.

## Author Contributions

Pengzhi Bu: Methodology, Original Draft Preparation; Ji Luo: Data Curation, Visualization; Chuanbiao Wen: Conceptualization, Supervision; Guangtao Pan: Writing – Review and Editing; Jing Xu: Conceptualization.

## Ethics Statement

The study was conducted in accordance with the Declaration of Helsinki, and approved by the Ethics Committee of the Affiliated Hospital of Chengdu University of Chinese Medicine (2023KL‐067, June 16, 2023).

## Consent

Informed consent was obtained from all subjects involved in the study. Written informed consent has been obtained from the patient(s) to publish this paper.

## Conflicts of Interest

The authors declare no conflicts of interest.

## Data Availability

The data that support the findings of this study are available on request from the corresponding author. The data are not publicly available due to privacy or ethical restrictions.

## References

[1] L. Li , “Expert Consensus on the Selection and Use of Home Beauty Devices for Anti‐Aging Facial Skin,” Chinese Journal of Dermatology and Venereology 37, no. 9 (2023): 977–982.

[2] M. L. W. Juhász , M. K. Levin , and E. S. Marmur , “A Review of Available Laser and Intense Light Source Home Devices: A Dermatologist's Perspective,” Journal of Cosmetic Dermatology 16, no. 4 (2017): 438–443. 28741866 10.1111/jocd.12371

[3] C. Ma , S. Piao , X. Yang , et al., “Research Progress on the Mechanisms of Aging and Acupuncture for Anti‐Aging,” Chinese Journal of Gerontology 39, no. 10 (2019): 2542–2546.

[4] S. Nassar , M. Assem , D. Mohamed , et al., “The Efficacy of Radiofrequency, Intense Pulsed Light and Carboxytherapy in Facial Rejuvenation,” Journal of Cosmetic and Laser Therapy 22, no. 6–8 (2020): 256–264. 33840336 10.1080/14764172.2021.1880598

[5] J. G. Labadie , S. Chilukuri , J. Cohen , et al., “Noninvasive Hands‐Free Bipolar Radiofrequency Facial Remodeling Device for the Improvement of Skin Appearance,” Dermatologic Surgery 49, no. 1 (2023): 54–59. 36533797 10.1097/DSS.0000000000003666 PMC9760459

[6] R. P. Arpini , M. S. Arpini , D. Pochmann , et al., “Non‐Invasive Radiofrequency Therapy Modulated Histone Acetylation Status Without Change Heat Shock Proteins in Healthy Women,” Journal of Plastic, Reconstructive & Aesthetic Surgery 74, no. 9 (2021): 2392–2442. 10.1016/j.bjps.2021.03.103 33947650

[7] D. H. Suh , H. J. Ahn , J. K. Seo , S. J. Lee , M. K. Shin , and K. Y. Song , “Monopolar Radiofrequency Treatment for Facial Laxity: Histometric Analysis,” Journal of Cosmetic Dermatology 19, no. 9 (2020): 2317–2324. 32319176 10.1111/jocd.13449

[8] G. Ablon , “Phototherapy With Light‐Emitting Diodes: Treating a Broad Range of Medical and Aesthetic Conditions in Dermatology,” Journal of Clinical and Aesthetic Dermatology 11, no. 2 (2018): 21. PMC5843358 29552272

[9] M. Cheung , A. S. Chan , and J. Yip , “Microcurrent Stimulation at Shenmen Acupoint Facilitates EEG Associated With Sleepiness and Positive Mood: A Randomized Controlled Electrophysiological Study,” Evidence‐Bbased Complementary and Alternative Medicine 2015, no. 1 (2015): 182837. 25767551 10.1155/2015/182837 PMC4342064

[10] X. Wang , “Traditional Chinese Medicine Treatment for Anti‐Aging Skin,” TMR Aging 2, no. 1 (2020): 26–30.

[11] W. Bo , T. Chunfeng , X. Qiong , et al., “Clinical Study on Puncturing EX‐HN5 (Taiyang) Towards ST4 (Dicang) and ST6 (Jiache) Treating Peripheral Facial Paralysis and Its Effect on Conduction Function of Facial Nerve,” International Journal of Clinical Acupuncture 30, no. 1 (2021): 1, 10.3103/S1047197921010026.

[12] Y. Moustafa , A. N. Kassab , J. El Sharnoubi , et al., “Comparative Study in the Management of Allergic Rhinitis in Children Using LED Phototherapy and Laser Acupuncture,” International Journal of Pediatric Otorhinolaryngology 77, no. 5 (2013): 658–665. 23394792 10.1016/j.ijporl.2013.01.006

[13] B. Wang , H. Y. Yang , T. Y. Liu , et al., “Photobiomodulation Effect of 635 Nm LED Irradiation on Kidney Yang Deficiency Model Rats,” Chinese Journal of Laser Medicine 19, no. 3 (2010): 142–147+202.

[14] K. Armstrong , R. Gokal , A. Chevalier , W. Todorsky , and M. Lim , “Microcurrent Point Stimulation Applied to Lower Back Acupuncture Points for the Treatment of Nonspecific Neck Pain,” Journal of Alternative and Complementary Medicine 23, no. 4 (2017): 2. 10.1089/acm.2016.0313 PMC5393407 28266863

[15] M. Cohen , E. Austin , N. Masub , et al., “Home‐Based Devices in Dermatology: A Systematic Review of Safety and Efficacy,” Archives of Dermatological Research 314, no. 3 (2022): 239–246. 33938981 10.1007/s00403-021-02231-0 PMC8918178

[16] P. Bu , R. Duan , J. Luo , T. Yang , N. Liu , and C. Wen , “Development of Home Beauty Devices for Facial Rejuvenation: Establishment of Efficacy Evaluation System,” Clinical, Cosmetic and Investigational Dermatology 17 (2024): 553–563, 10.2147/CCID.S449599. 38476342 PMC10929553

[17] Y. Ma , N. Wang , K. Li , H. Liang , J. Bai , and X. Ji , “Effect of Geometric Parameters of Electrodes on Skin Heating for the Design of Non‐Ablative Radiofrequency Device,” Skin Research and Technology 29, no. 10 (2023): e13472. 37881053 10.1111/srt.13472 PMC10560826

[18] K. T. Mellody , E. J. Bradley , B. Mambwe , et al., “Multifaceted Amelioration of Cutaneous Photoaging by (0.3%) Retinol,” International Journal of Cosmetic Science 44, no. 6 (2022): 625–635. 35778881 10.1111/ics.12799 PMC9826105

[19] A. L. S. Oliveira , D. Valente , H. R. Moreira , et al., “Effect of Squalane‐Based Emulsion on Polyphenols Skin Penetration: Ex Vivo Skin Study,” Colloids and Surfaces B: Biointerfaces 218 (2022): 112779. 35994992 10.1016/j.colsurfb.2022.112779

[20] M. S. Kim , “A Study on Cosmetic Acupuncture Through Anatomy and Physiology Interpretation,” Korean Journal of Acupuncture 30, no. 3 (2013): 171–177.

[21] R. H. Lv , W. P. Xin , and X. Wang , “The Effect of Acupoint Filler Injection on Facial Rejuvenation Based on Meridian Theory,” Chinese Journal of Aesthetic Medicine 30, no. 11 (2021): 103–106.

[22] R. H. Lv , “The Application Effect of Avelan Combined With ACO Injection Technology in Facial Rejuvenation,” Medical Aesthetics and Cosmetology 31, no. 19 (2022): 1–3.

[23] Z. B. Yang , J. C. Shen , Y. D. Wang , et al., “Study on the Central Response Mechanism of Electroacupuncture Treatment for Gastric Ulcers Using 1H NMR Metabolomics,” Chinese Journal of Traditional Chinese Medicine 32, no. 7 (2017): 3191–3195.

[24] D. Amorim , I. Brito , A. Caseiro , et al., “Electroacupuncture and Acupuncture in the Treatment of Anxiety ‐ A Double Blinded Randomized Parallel Clinical Trial,” Complementary Therapies in Clinical Practice 46 (2022): 101541. 35124475 10.1016/j.ctcp.2022.101541 PMC9760487

[25] C. Wang , W. L. Xu , G. W. Li , et al., “Impact of Acupuncture on Sleep and Comorbid Symptoms for Chronic Insomnia: A Randomized Clinical Trial,” Nature and Science of Sleep 13 (2021): 1807–1822. 10.2147/NSS.S326762 PMC8519353 34675728

[26] M. J. Kim , S. Lee , and S. N. Kim , “Effects of Acupuncture on Gastrointestinal Diseases and Its Underlying Mechanism: A Literature Review of Animal Studies,” Frontiers in Medicine 10 (2023): 1167356. 37351066 10.3389/fmed.2023.1167356 PMC10282137

[27] M. H. Gold , J. Biron , L. Levi , et al., “Safety, Efficacy, and Usage Compliance of a Home‐Use Device Utilizing RF and Light Energies for Treating Periorbital Wrinkles,” Journal of Cosmetic Dermatology 16, no. 1 (2017): 95–102. 27910259 10.1111/jocd.12299

